# miR-22-3p and miR-30e-5p Are Associated with Prognosis in Cervical Squamous Cell Carcinoma

**DOI:** 10.3390/ijms23105623

**Published:** 2022-05-17

**Authors:** Ah-Young Kwon, Ju-Yeon Jeong, Hyun Park, Sohyun Hwang, Gwangil Kim, Haeyoun Kang, Jin-Hyung Heo, Hye Jin Lee, Tae-Heon Kim, Hee Jung An

**Affiliations:** 1 Department of Pathology, CHA University College of Medicine, Seongnam 13496, Gyeonggi-do, Korea; kwonahyoung@gmail.com (A.-Y.K.); blissfulwin@chamc.co.kr (S.H.); blacknw@cha.ac.kr (G.K.); hykang@cha.ac.kr (H.K.); sacrum77@chamc.co.kr (J.-H.H.); ra41041@chamc.co.kr (H.J.L.); ice_t69@cha.ac.kr (T.-H.K.); 2 Institute of Clinical Research, CHA University College of Medicine, Seongnam 13496, Gyeonggi-do, Korea; 3 CHA Future Medical Research Institute, CHA University College of Medicine, Seongnam 13496, Gyeonggi-do, Korea; dufakthd@chamc.co.kr; 4 Department of Gynecological Oncology, CHA University College of Medicine, Seongnam 13496, Gyeonggi-do, Korea; p06162006@cha.ac.kr

**Keywords:** miRNA, uterine cervix, squamous cell carcinoma, metastasis, miR-30e-5p, miR-22-3p

## Abstract

Alteration in expression of miRNAs can cause various malignant changes and the metastatic process. Our aim was to identify the miRNAs involved in cervical squamous cell carcinoma (SqCC) and metastasis, and to test their utility as indicators of metastasis and survival. Using microarray technology, we performed miRNA expression profiling on primary cervical SqCC tissue (n = 6) compared with normal control (NC) tissue and compared SqCC that had (SqC-M; n = 3) and had not (SqC-NM; n = 3) metastasized. Four miRNAs were selected for validation by qRT-PCR on 29 SqC-NM and 27 SqC-M samples, and nine metastatic lesions (ML-SqC), from a total of 56 patients. Correlation of miRNA expression and clinicopathological parameters was analyzed to evaluate the clinical impact of candidate miRNAs. We found 40 miRNAs differentially altered in cervical SqCC tissue: 21 miRNAs were upregulated and 19 were downregulated (≥2-fold, *p* < 0.05). Eight were differentially altered in SqC-M compared with SqC-NM samples: four were upregulated (miR-494, miR-92a-3p, miR-205-5p, and miR-221-3p), and four were downregulated (miR-574-3p, miR-4769-3p, miR-1281, and miR-1825) (≥1.5-fold, *p* < 0.05). MiR-22-3p might be a metastamiR, which was gradually further downregulated in SqC-NM > SqC-M > ML-SqC. Downregulation of miR-30e-5p significantly correlated with high stage, lymph node metastasis, and low survival rate, suggesting an independent poor prognostic factor.

## 1. Introduction

Cervical cancer is one of the most common cancers in women worldwide. According to the Surveillance, Epidemiology, and End Results Program (SEER), 6.5 per 100,000 new cases of cervical cancer are diagnosed per year, and 6.8 per 100,000 women per year die from it. Squamous cell carcinoma (SqCC) is the most common type of cervical cancer and is well known to be linked to human papillomavirus (HPV) infection. SqCC can be detected in the early stages by cervical screening; however, the early detection of metastasis is currently difficult. The five-year survival rate for patients with SqCC decreases according to the extent of the disease, as follows: confined to a local site (91.5%), regional lymph node metastasis (57.4%), and distant metastasis (16.5%) [1]. Therefore, identifying lymph node or distant metastasis in cervical cancer is important, not only for predicting prognosis, but also for choosing the most appropriate treatment strategy. For example, advanced disease with distant metastasis is treated with chemotherapy and/or radiation with or without lymph node dissection, possibly also including molecular targeted therapy [1].

While it is now well-established that the main cause of cervical cancer is oncogenic HPV infection [2], the presence of a persistent infection with a high oncogenic risk HPV genotype is not sufficient to induce cervical carcinogenesis; HPV induces cervical dysplasia and carcinoma in only 10% of infected women. Multiple genetic and epigenetic alterations after HPV infection, e.g., miRNA, are known to be required for the development of cervical SqCC from squamous epithelium, through dysplasia, to carcinoma [2].

MicroRNAs (miRNAs) are small non-coding RNA sequences consisting of 18–25 nucleotides [3,4] implicated in post-transcriptional silencing of genes by their binding to the 3′-untranslated region of target messenger RNAs (mRNAs) [5,6]. Their effects are considered to be epigenetic alterations. The dysregulated expression of various miRNAs has been reported in many human cancers, including cervical SqCC, for which specific miRNA expression profiling has been performed in several previous studies [7,8,9,10]; however, the role of miRNAs in the metastasis of cervical SqCC remains largely unexplored.

In the present study, we aim to find the miRNAs involved in the progression of SqCC, especially the metastatic process. First, we identified miRNAs differentially expressed in cervical SqCC compared with normal squamous epithelium (NC) by microarray expression profiling. To identify the miRNA candidates associated with the metastasis in SqCC, miRNA expression profile was compared between SqCCs that had (SqC-M) or had not (SqC-NM) metastasized, and between SqCCs and metastatic lesions of lymph nodes or distant area (ML-SqC). Then, the expression of the most relevant miRNAs was validated by quantitative real-time polymerase chain reaction (qRT-PCR). Finally, the correlation between the miRNA expression levels and clinicopathologic factors was investigated. Through this study, we identified candidate miRNAs associated with the metastatic process, and assessed their utility as biomarkers for prediction of metastasis and prognosis.

## 2. Results

### 2.1. Differential miRNA Profiles on miRNA Microarray

**Figure 1:**
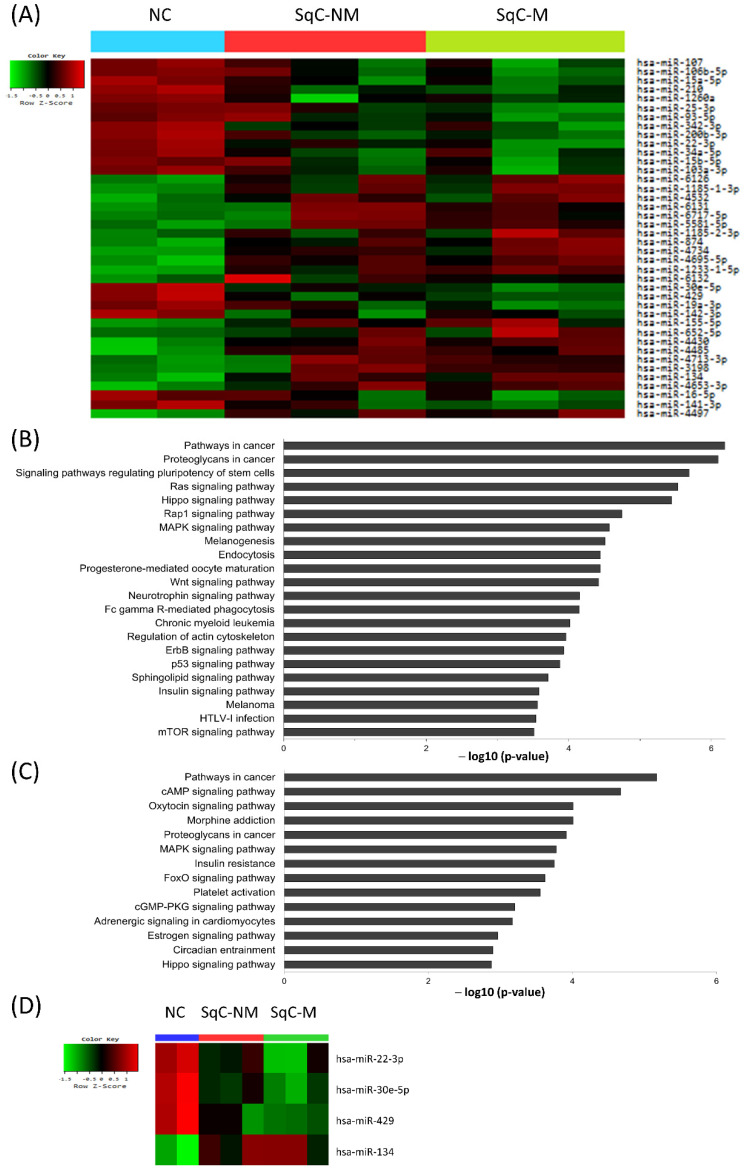
(**A**) Hierarchical clustering of the 40 miRNAs differentially regulated between normal epithelium (n = 2) and cervical squamous cell carcinoma (n = 6). (**B**) KEGG pathway annotation analysis of target genes of the significantly dysregulated 40 miRNAs in 6 cases of cervical SqCC compared with NC, and (**C**) KEGG pathway annotation analysis of target genes of the significantly dysregulated 8 miRNAs in 3 cases of SqC-M compared with SqC-NM (*p* < 0.05). The enrichment score of each pathway is expressed as ─log (*p*-value). (**D**) Hierarchical clustering of the four selected miRNAs. NC, normal squamous epithelium; SqC-NM, squamous cell carcinoma without metastasis; SqC-M, squamous cell carcinoma with metastasis. The color indicates the signal value, from red (upregulation) to green (downregulation).

To identify miRNAs that are associated with the development and metastasis in SqCCs, we performed miRNA microarray analysis. Microarray analysis identified 40 miRNAs with levels that were statistically significantly altered by more than 2-fold (*p* < 0.05) in SqCC relative to NC tissue, including 21 upregulated miRNAs and 19 downregulated miRNAs (Table 1). The microarray data presented in this study were prepared according to the Minimum Information About Microarray Experiment (MIAME) recommendations and are available through the NCBI GEO, with accession number GSE112743. Hierarchical clustering of 40 miRNAs in NC and SqCC tissues is shown in Figure 1A.

To access the functional implication of these 40 significantly dysregulated miRNAs in SqCC compared with NC tissue, we predicted the target genes of these miRNAs using TargetScan 7.1 (Figure 1B). A total of 1850 genes were found to be associated with the 40 miRNAs. Functional pathways were ranked according to the number of gene members in the list. ‘Pathways in cancer’ and ‘proteoglycans in cancer’ were the most significantly enriched pathways. Twelve signaling pathways (‘signaling pathways regulating pluripotency of stem cells’; Ras, Hippo, Rap1, MAPK, Wnt, neurotrophin, ErbB, p53, sphingolipid, insulin, and mTOR signaling pathways) were highly ranked. In addition, pathways associated with specific cancers (chronic myeloid leukemia and melanoma) and cellular metabolism (endocytosis and actin cytoskeleton) were also identified.

When we compared the miRNA expression profile of SqC-M relative to SqC-NM to identify the miRNAs implicated in the metastasis of these tumors, we identified eight miRNAs with a fold change difference more than 1.5-fold (*p* < 0.05) in SqC-M relative to SqC-NM. Four miRNAs were upregulated: miR-574-3p (2.29-fold), miR-4769-3p (4.19-fold), miR-1281 (4.48-fold), and miR-1825 (4.57-fold), and four miRNAs were downregulated: miR-221-3p (0.64-fold), miR-205-5p (0.49-fold), miR-92a-3p (0.42-fold), and miR-494 (0.32-fold). To predict the functional role of these miRNAs, we then examined the target genes of these eight miRNAs dysregulated in SqC-M compared with SqC-NM using TargetScan 7.1 (Figure 1C). A total of 3080 genes were selected and used for the functional annotation analysis. ‘Pathways in cancer’ was also ranked highest, followed by ‘cAMP signaling pathway’ and ‘oxytocin signaling pathway’, which are both associated with Ca++ influx and efflux and cellular locomotion.

Among these miRNAs, we selected four miRNAs (miR-30e-5p, -22-3p, -429, and -134) to be validated by qRT-PCR in SqC-NM, SqC-M, and ML-SqC tissue according to the following criteria: (1) increased (miR-22-3p, -30e-5p, and -429) or decreased (miR-134) expression tendencies in the NC tissue to SqC-NM to SqC-M, and (2) a relatively consistent pattern of expression within a group. Figure 1D shows the hierarchical clustering heatmap of the four selected miRNAs. Other miRNAs did not show a distinct and definite pattern of expression.

### 2.2. Validation of miRNA Expression by qRT-PCR

To identify relevant miRNAs for biomarkers predicting metastasis, the four miRNAs (miR-22-3p, -30e-5p, -429, and -134) were selected according to the criteria described in the Materials and Methods section. They were validated by qRT-PCR in 29 cases of SqC-NM, 27 cases of SqC-M, 9 cases of ML-SqC, and ten NCs.

**Figure 2:**
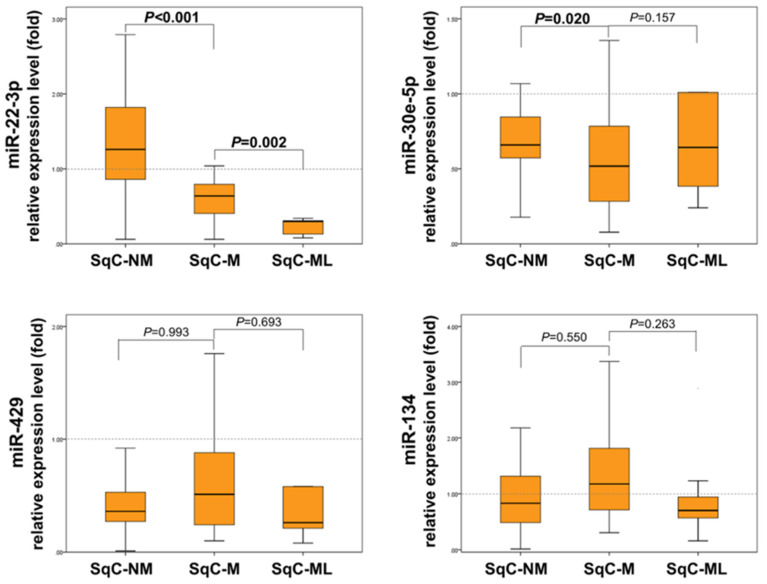
Box and whisker plots of miR-22-3p, miR-30e-5p, miR-429, and miR-134 expression levels in primary cervical SqCC without metastasis (SqC-NM; n = 29), primary cervical SqCC with metastasis (SqC-M; n = 27), and metastatic lesions from cervical SqCC (SqC-ML; n = 9) relative to the level of expression in normal squamous epithelium, by qRT-PCR. MiR-22-3p shows a tendency for downregulation in SqC-NM to SqC-M to SqC-ML (*p* < 0.001 and *p* = 0.002, respectively). MiR-30e-5p shows significant downregulation from SqC-NM to SqC-M (*p* = 0.020). MiRNA-134 and miRNA-429 showed no significant differences in expression levels between the different types of tissue.

The raw data is provided in the Supplementary Table S1. The expression levels of the selected miRNAs in the 65 cases of SqCC (SqC-NM, SqC-M, and ML-SqC) are shown in Table 2. Expression of miR-30e-5p and miR-429 were significantly decreased in cervical SqCC compared with NC tissue (0.86-fold, *p* = 0.002 and 0.70-fold, *p* < 0.001, respectively). When compared among the groups of SqC-NM, SqC-M, and ML-SqC, miR-22-3p exhibited a decreasing expression pattern during the transition from SqC-NM to SqC-M to ML-SqC, with statistical significance (Figure 2). Compared with the NC tissue, the mean expression level of miR-22-3p was 1.61-fold in SqC-NM, 0.82-fold in SqC-M, and 0.27-fold in ML-SqC. The expression of miR-22-3p was significantly downregulated in SqC-M compared with SqC-NM (*p* < 0.001, Mann–Whitney test), and in SqC-M compared with ML-SqC (*p* = 0.002, Mann–Whitney test). The difference in miR-30e-5p expression was not significant among these three groups (*p* = 0.054, Kruskal–Wallis test); however, it was significantly downregulated in SqC-M compared with SqC-NM (*p* = 0.020, Mann–Whitney test). MiR-429 and miR-134 did not show significant differences in expression among these three groups.

### 2.3. Association between miRNA Expression and Clinicopathologic Prognostic Factors

To assess the clinical impact of the four selected miRNAs, we evaluated the association between their expression levels and clinicopathologic parameters of cervical SqCC (Table 3). Nodal metastasis was significantly correlated with downregulation (<0.5-fold) of miR-22-3p (*p* = 0.020, chi-square test) and downregulation (<0.4-fold) of miR-30e-5p (*p* = 0.042, chi-square test). Downregulation of miR-30e-5p was also significantly correlated with lymphovascular invasion (*p* = 0.025, Fisher’s exact test) and high clinical stage (stages II–IV; *p* = 0.038, chi-square test). The upregulation of miR-134 (≥1.5-fold) was significantly correlated with lymphovascular invasion and recurrence (*p* = 0.043 and 0.019, respectively, Fisher’s exact test).

### 2.4. HPV Infection Pattern and Its Association with miRNA Expression

We also confirmed the association between these miRNAs and HPV, the cause of SqCC. Among the 56 patients with cervical SqCC included in the present study, HPV infection was shown in all cases. HPV infection with a high-risk genotype (16/18/31/33/35/45/52/56/58) was revealed in all 56 patients, 35 of whom had HPV 16. Seven cases showed multiple HPV infection, including HPV 16 and 58 (n = 3); HPV 16 and 31 (n = 1); HPV 16 and 34 (n = 1); HPV 18 and 6 (n = 1); and HPV 18, 44, and other type (n = 1). We analyzed the correlation between miRNA expression and HPV types. HPV 16 infection was significantly correlated with upregulation (≥1.8-fold) of miR-22-3p (*p* = 0.039, Fisher’s exact test; Table 4), whereas no association with HPV genotypes was revealed for the other three miRNAs.

### 2.5. Survival and Multivariate Cox Analysis

**Figure 3:**
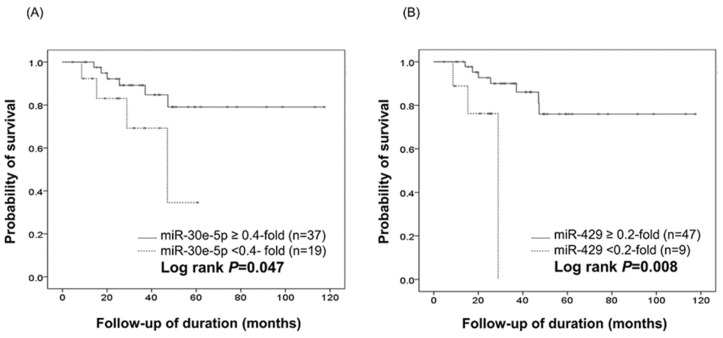
Kaplan–Meier survival curves according to miR-30e-5p (**A**) and miR-429 (**B**) expression in 56 cases of cervical SqCC. Downregulation of miR-30e-5p (<0.4-fold relative to the normal epithelium (NC) level) and miR-429 (<0.2-fold relative to the NC level) each correlates with shorter overall survival with statistical significance (log rank = 0.047 and 0.008, respectively).

Overall survival (OS) analysis of the 56 patients, regardless of metastasis, was performed according to the expression levels of each of the four selected miRNAs. During the follow-up period, ten patients died of disease, and all were from the SqC-M group. Kaplan–Meier survival analysis according to miR-30e-5p and miR-429 expression (Figure 3) demonstrated that downregulation of miR-30e-5p (<0.4-fold) and miR-429 (<0.2-fold) was significantly associated with shorter OS (miR-30e-5p, OS: 69.2% vs. 86.0%, 28.1 ± 15.7 months vs. 42.0 ± 26.7 months, log rank = 0.047; miR-429, OS: 66.7% vs. 85.1%, 20.5 ± 7.6 months vs. 42.2 ± 25.9 months, log rank = 0.008). MiR-22-3p and miR-134 did not show significant difference in OS analysis (Figure S1).

A multivariate Cox regression analysis was performed for clinicopathologic parameters and expression levels of miR-30-5p and miR-429 (Table 5). A low expression level (<0.4-fold compared with the NC) of miR-30e-5p was significantly associated with a low survival rate (*p* = 0.040, hazard ratio = 289.66), indicating that downregulation of miR-30e-5p could be an independent poor prognostic factor in cervical SqCC. In this analysis, as expected, age (≥60 years) and distant metastasis were also poor prognostic factors. Multivariate Cox regression analysis failed to confirm miR-429 as a prognostic factor.

## 3. Discussion

Metastasis is related to multiple biological processes and genes, including migration, invasion, resistance to apoptosis, and angiogenesis. The expression of metastasis-associated genes could be regulated by epigenetic effectors, such as DNA methylation, histone modification, and miRNAs [11]. MiRNAs, small non-coding single-stranded RNAs, base-pair with complementary nucleotide sequences of target mRNAs, inhibiting the mRNA, and thereby regulating gene expression at the post-transcriptional level. Many miRNAs are expressed differentially in many neoplasms [12]. Several studies have identified an association between miRNA expression and tumor metastasis, as well as tumor development. Whereas miRNAs regulating tumorigenicity are designated as oncomiRs or tumor suppressor miRNAs, miRNAs related to invasion and metastasis are known as metastamiRs [11]. They have pro- or anti-metastatic effects, which promote or suppress various steps in migration and metastasis of cancer cells [13].

Cervical SqCC is one of the most common gynecological malignancies worldwide [14]. Through multiple studies, HPV has been identified as a cause in cervical carcinoma, and in particular the genotypes HPV 16 and 18 [15]. HPV infection and constitutive expression of E6 and E7 viral oncogenes interacts with some cellular proteins, such as c-MYC, and leads to degradation of p53 and pRB proteins [16,17,18]. Along with viral oncogenes, a series of epigenetic factors, including miRNAs, have been identified in cervical carcinogenesis. In cervical cancer cells, the expression of miR-21 is significantly higher, and that of miR-143, miR-196b, and the miRNA precursor let-7c is significantly lower [19]. Furthermore, many studies report altered expression of many miRNAs in cervical SqCC cell lines or primary tissues [7,8,20,21]. We identified 40 miRNAs associated with cervical SqCC significantly by microarray: increased up to 16.5-fold and decreased up to 7-fold (Table 1).

Metastasis-associated miRNAs were also of interest. Regarding miRNAs associated with metastasis in cervical SqCC, there are some reports that increased miR-20a, miR-127, miR-200, miR-224, and miR-375, and decreased miR-203 and miR-214 expression are significantly associated with metastasis [7,22,23,24,25,26]. Another study reported that downregulation of let-7c, miR-100, miR-125b, miR-143, miR-145, and miR-199a-5p is significantly associated with lymph node metastasis [27]. Chen et al. investigated serum miRNAs in patients with metastatic SqCC and found high expression of miR-20a, miR-1246, miR-2392, miR-3147, miR-3162-5p, and miR-4484 [28]. Moreover, miR-9 was found to be increased in metastatic SqCC tissue in a mouse model [29], and downregulation of miR-29a induced SqCC development and metastasis in an in vitro study [30]. Additionally, other studies revealed specific miRNAs associated with metastasis-related pathways, such as epithelial-mesenchymal transition, metalloproteinases, and angiogenesis [31]. These studies only analyzed a few individual miRNAs; however, recently a high-throughput analysis using miRNA microarray to explore miRNA expression profiling in cancers with lymph node metastasis detected 39 differentially expressed miRNAs [32]. Using qRT-PCR on ten samples, the study verified that miR-490-5p, miR-323-3p, miR-657, miR-126, miR-96, and miR-144 were differentially expressed in SqCC with lymph node metastasis compared with SqCC without.

However, there has been no previous study examining the correlation between miRNA expression profiles and clinical outcome. In the present study, we performed high-throughput analysis using miRNA microarray and verified miRNA expression by qRT-PCR, in SqCCs without and with metastasis and in the metastatic lesions, with the aim of identifying putative metastamiRs. Of the 40 differentially expressed miRNAs initially identified by comparing the profiles of SqCC and NC tissue, the downregulation of miR-142-3p, miR-16-5p, and miR-34a, and upregulation of miR-155-5p were in accordance with the findings of previous studies [8,33,34,35]. In addition, we found that the dysregulated miRNAs in SqCC were associated not only with multiple signaling pathways, but also with pathways involved in cellular metabolism such as endocytosis, phagocytosis, and regulation of the actin cytoskeleton according to the Kyoto Encyclopedia of Genes and Genomes (KEGG) pathway database. These metabolic and signaling pathways may play an important role in the development of cervical SqCC. On the other hand, the cAMP and oxytocin signaling pathways were miRNA targets in the SqC-M tissue group, suggesting a role for them in metastasis of SqCC.

In this study, of the four miRNAs selected for validation, miR-22-3p was upregulated in SqC-NM compared with NC tissue and showed a significantly decreasing expression pattern from SqC-NM to SqC-M to ML-SqC, suggesting that it is a metastamiR, functioning in metastatic progression rather than carcinogenesis. We also report here that downregulation of miR-22-3p was significantly correlated with nodal metastasis. In accordance with our finding, a low miR-22 expression level has been shown to significantly correlate with metastasis in osteosarcoma, epithelial ovarian cancer, hepatocellular carcinoma, and colorectal cancer [36,37,38,39,40]. In addition, Yang et al. showed that miR-22 expression inhibits cell migration and invasion [41], and in gastric and breast cancers, miR-22 expression suppresses cell invasion and metastasis in vitro and in vivo [42,43]. Taken together, miR-22 may function as a metastamiR in many human cancers, including cervical SqCC. The target of miR-22-3p is known as eIF4E-binding protein 3 (eIF4EBP3), a component of the PI3K/AKT pathway [44,45], which plays an important role in tumorigenesis and metastasis of many cancers.

Interestingly, HPV 16 infection was significantly correlated with upregulation of miR-22-3p in the present study, suggesting that miR-22-3p expression is mechanistically associated with HPV infection, so called HPV core miRNAs [46]. This is the first report of their association. Considering the previous study that HPV-positive cervical SqCCs had a better prognosis than the HPV-negative cancers [47], it is understandable that downregulation of miR-22-3p, one of HPV core miRNAs, was found to be related to the metastasis of cervical SqCC.

In the present study, the expression levels of miR-30e-5p and miR-134 did not show significant differences among SqC-NM, SqC-M, and ML-SqC. However, downregulation of miR-30e-5p and upregulation of miR-134 correlated with higher stage, more frequent nodal metastasis, lymphovascular invasion, and recurrence. Downregulation of miR-30e-5p, in particular, was related to shorter survival in cervical SqCC. This result is in accordance with other reports showing that its expression is negatively related to OS in lung and gastrointestinal cancers [48,49]. Taken together, miR-30e-5p is an independent prognostic factor in cervical SqCC by multivariate analysis, suggesting that miR-30e-5p functions as a tumor suppressor in cervical SqCC as in various other tumors. This is supported by the study of Zhang et al. that miR-30e-5p inhibits metastasis and angiogenesis in squamous cell carcinoma cells of the head and neck, and down-regulation of miR-30e-5p was associated with poorer overall survival [50]. Therefore, miR-30e-5p is a candidate for a biomarker of tumor prognosis, and can be a therapeutic target in various metastatic tumors.

## 4. Materials and Methods

### 4.1. Cervical Tissue Specimens

Formalin-fixed paraffin-embedded (FFPE) cervical tissues from patients with cervical SqCC seen at the CHA Bundang Medical Center between 1 January 2005 and 31 March 2015 were retrieved from the archive. Of the 321 patients diagnosed with cervical SqCC, samples from 56 patients were included in this study, according to the exclusion criteria: (1) only biopsied specimen with uncertain pathologic stage, (2) no systemic evaluation, (3) histologically not confirmed metastasis although clinically suspected, (4) insufficient tissue for RNA extraction, or (5) any anticancer therapy before the tissue was taken. Patients were divided into two groups according to whether they had SqC-M or SqC-NM. SqC-M patients were enrolled only if metastasis could be evaluated pathologically from the lymph node dissection or excised distant lesion. The SqC-NM group included patients without evidence of metastasis, both pathologically by pelvic lymph node dissection and radiologically. The ML-SqC tissues were obtained from metastatic lesions, such as lymph nodes, bladder, or peritoneum, of nine patients with SqC-M, randomly selected. For controls, ten NC tissues were obtained from patients with benign uterine disease (e.g., leiomyoma or adenomyosis) without history of cervical dysplasia or an abnormal Pap smear. Clinicopathologic data, including age, International Federation of Gynecology and Obstetrics (FIGO) stage, presence of metastasis, and survival period, were obtained from medical records, pathologic reports, and the Korea Central Cancer Registry. The patient characteristics are summarized in Table S2. This study was approved by the Ethics Committee of CHA Bundang Medical Center (CHAMC 2020-04-048-003).

### 4.2. HPV Genotyping

A PCR-based DNA microarray, HPVDNAChip (AG Bio Diagnostics Co., Seoul, Korea), was used for HPV genotyping according to the manufacturer’s protocol. HPV genotypes classified as being of high (16/18/31/33/35/39/45/51/52/56/58/59/66/68/69) and of low (6/11/34/40/42/43/44) oncogenic risk could be identified using the HPVDNAChip.

### 4.3. MiRNA Extraction and Microarray Hybridization

As miRNAs can remain stable in FFPE blocks for more than a decade, archival FFPE tissues are suitable for miRNA analysis. RNA isolation from FFPE tissues was performed using the RNeasy FFPE Kit (QIAGEN, Hilden, Germany) according to the manufacturer’s protocol. The purity and concentration of RNA were confirmed using a NanoDrop ND-1000 (NanoDrop Technologies, Wilmington, DE, USA).

MiRNA expression profiles by miRNA microarray were assessed in six cases of SqCC, including three SqC-NMs and three SqC-Ms, and in two NCs. The six cases of SqCC were selected from the 56 SqCC cases to be introduced in the qRT-PCR assay, described in the later. We selected the samples for microarray study in which the amount of extracted RNAs were enough to examine. The patients for microarray study were as follows: 3 patients of non-metastatic squamous cell carcinoma (Sq-NM)—2 patients with stage IB and one patient with stage IIA; 3 patients of squamous cell carcinoma with metastasis (SqC-M)—two patients with stage IIIB and one patient with stage IVB. We used the SurePrint G3 Custom miRNA Microarray platform (Agilent Technologies, Santa Clara, CA, USA). Microarray analysis was performed as follows: briefly, desalted labeled RNA was prepared from 100 ng total RNA using the Agilent miRNA Complete Labeling and Hyb Kit with Cyanine 3-pCp (Cy3) (Agilent Technologies). Labeled miRNAs were hybridized to the miRNA expression microarray according to the manufacturer’s protocol. The arrays were washed and scanned using the Agilent microarray scanner (G2600D, Agilent Technologies). Raw data were extracted using Agilent Feature Extraction software v11.0.1.1 (Agilent Technologies). After filtering, the selected miRNA signal value was then transformed by logarithm method and normalized by the quantile method. The comparative analysis between SqCCs and NCs, and between SqC-NM and SqC-M samples, was based on fold change. Data analyses and assessment of differentially expressed genes were performed using R statistical software v.3.1.2 (The R Foundation, Vienna, Austria). MiRNAs were considered to be differentially expressed between SqCCs and NCs if the difference reached at least 2-fold, and between SqC-M and SqC-NM tissues if the difference reached at least 1.5-fold.

### 4.4. MiRNA Target Prediction and Functional Annotation Analysis

MiRNAs differentially expressed in SqCC compared with the NCs, and in SqC-M compared with SqC-NM, based on microarray data, were selected. TargetScan 7.1 (Whitehead Institute, Cambridge, MA, USA) software was used to identify the target genes predicted for each miRNA. Of all the target genes for the 40 miRNAs, the genes that were repeated five or more times were listed. The target gene list was submitted to the Database for Annotation, Visualization, and Integrated Discovery (DAVID) website and annotated using the KEGG pathway enrichment analysis database. The Benjamini–Hochberg adjusted *p*-value < 0.05 was chosen as the cutoff criteria.

### 4.5. Validation by qRT-PCR

Total RNA was extracted from validation sets consisting of 29 cases of SqC-NM, 27 cases of SqC-M, 9 cases of ML-SqC, and ten NCs, and reverse transcribed using the TaqMan MicroRNA Reverse Transcription Kit (Applied Biosystems, Foster City, CA, USA) according to the manufacturer’s instructions. We used a pre-designed commercial assay of TaqMan probes from Applied Biosystems: miR-429 (ID:001024), miR-22-3p (ID:000398), miR-30E-5p (ID:002223), and miR-134-5p (ID:000459), and RNU48 (ID:001006) was used as an internal control on a Bio-rad CFX96 Real-Time PCR System (Bio-Rad, Hercules, CA, USA) according to the manufacturer’s instructions. RT-PCR was performed using the Bio-Rad CFX96 Real-Time PCR Detection System according to the manufacturer’s instructions. All PCR reactions were run twice, and the relative quantification of miRNA expression was calculated with the 2−ΔΔCt method. RNU48 was used as an endogenous control, and the expression levels of each miRNA in cancerous tissues were presented as fold change compared with those in the NCs.

### 4.6. Statistical Analysis

Expression levels of miRNAs in cancer cells and NCs were analyzed by the Kruskal–Wallis test and Mann–Whitney test. The chi-square test or Fisher’s exact test was used to analyze the correlation between miRNA expression levels and clinicopathological data. The survival analysis according to expression of each miRNA was performed using Kaplan–Meier curves, and the differences were assessed by the log-rank test. Multivariate Cox regression analysis was performed to assess associations between miRNA expression and survival. *p* < 0.05 was considered statistically significant. Statistical analysis was performed using the SPSS 22.0 package (SPSS, Chicago, IL, USA).

## 5. Conclusions

We found 40 miRNAs differentially expressed in cervical SqCC by microarray, four of which were further analyzed by qRT-PCR for their roles as putative metastamiRs. We found that miR-22-3p functions as a metastamiR, as its expression gradually decreased in tissue samples representing the stages from non-metastatic primary tumor to metastatic primary tumor to metastatic lesion. In addition, downregulation of miR-30e-5p was significantly correlated with high stage, lymph node metastasis, and low survival rate.

Taken together, downregulation of miR-22-3p is involved in the metastatic process, and downregulation of miR-30e-5p is an independent poor prognostic factor in cervical SqCC.

## Figures and Tables

**Table 1:** MiRNAs significantly upregulated (A) or downregulated (B) by more than 2-fold in cervical squamous cell carcinoma compared with normal epithelium.

**A. Upregulated miRNAs**			**B. Downregulated miRNAs**		
**miRNAs**	**Fold Change**	***p*-Value**	**miRNAs**	**Fold Change**	***p*-Value**
hsa-miR-134	2.019	0.023	hsa-miR-210	−7.229	<0.001
hsa-miR-1185-2-3p	2.066	0.017	hsa-miR-142-3p	−5.740	0.002
hsa-miR-4532	2.093	0.027	hsa-miR-1260a	−5.345	0.004
hsa-miR-652-5p	2.142	0.024	hsa-miR-429	−5.062	0.025
hsa-miR-155-5p	2.311	0.002	hsa-miR-15a-5p	−4.607	0.004
hsa-miR-6717-5p	2.478	0.011	hsa-miR-19a-3p	−4.577	0.005
hsa-miR-6131	2.583	0.006	hsa-miR-141-3p	−3.743	0.011
hsa-miR-1185-1-3p	2.804	0.003	hsa-miR-30e-5p	−3.593	0.015
hsa-miR-6126	2.833	0.003	hsa-miR-16-5p	−3.470	0.043
hsa-miR-4734	2.997	<0.001	hsa-miR-200b-3p	−3.142	0.002
hsa-miR-3198	3.085	0.005	hsa-miR-103a-3p	−3.044	0.004
hsa-miR-4713-3p	3.125	0.011	hsa-miR-107	−2.998	0.005
hsa-miR-5581-5p	3.189	0.013	hsa-miR-15b-5p	−2.899	0.015
hsa-miR-874	3.220	0.004	hsa-miR-22-3p	−2.618	0.003
hsa-miR-6132	3.267	0.027	hsa-miR-106b-5p	−2.426	0.008
hsa-miR-1233-1-5p	4.516	<0.001	hsa-miR-34a-5p	−2.290	0.003
hsa-miR-4485	4.679	0.032	hsa-miR-342-3p	−2.226	<0.001
hsa-miR-4695-5p	5.950	0.017	hsa-miR-93-5p	−2.123	0.020
hsa-miR-4497	6.501	0.009	hsa-miR-25-3p	−2.088	0.017
hsa-miR-4430	6.522	0.042			
hsa-miR-4653-3p	16.451	0.022			

**Table 2:** The relative expression level of the four miRNAs in cervical squamous cell carcinoma compared with normal squamous epithelium, and in each group of cervical squamous cell carcinoma without metastasis, with metastasis, and metastatic squamous cell carcinoma, by qRT-PCR.

miRNA	Cervical SqC (n = 65)	*p*-Value	Cervical SqC without Metastasis (n = 29)	Cervical SqC with Metastasis (n = 27)	Metastatic SqC from Cervix (n = 9)	*p*-Value
miR-22-3p	1.079 ± 1.191	0.228	1.609 ± 1.415	0.819 ± 0.881	0.266 ± 0.181	<0.001 *
miR-30e-5p	0.857 ± 0.842	0.002 *	0.891 ± 0.672	0.651 ± 0.600	1.370 ± 1.577	0.054
miR-429	0.696 ± 0.839	<0.001*	0.634 ± 0.752	0.693 ± 0.780	0.900 ± 1.270	0.744
miR-134	1.264 ± 1.319	0.429	1.359 ± 1.651	1.277 ± 0.902	0.930 ± 0.782	0.407

SqC, squamous cell carcinoma (*, *p* < 0.05).

**Table 3:** Correlation between clinicopathologic factors and miRNA expression.

	miR-420 ^§^		miR-22-3p ^§^		miR-30e-5p ^§^		miR-134 ^§^	
	Down-Regulation	*p*-Value	Down-Regulation	*p*-Value	Down-Regulation	*p*-Value	Up-Regulation	*p*-Value
**Age**								
<60	9/49 (18.4%)	0.583	13/49 (26.5%)	0.666	13/49 (26.5%)	0.182	36/49 (73.5%)	0.182
≥60	0/7 (0.0%)		1/7 (14.3%)		0/7 (0.0%)		3/7 (42.9%)	
**Clinical stage**								
I	4/27 (14.8%)	1.000	5/27 (18.5%)	0.280	3/27 (11.1%)	0.038 *	20/27 (74.1%)	0.487
II, III and IV	5/29 (17.2%)		9/29 (31.0%)		10/29 (34.5%)		19/29 (65.5%)	
**Nodal metastasis**								
absent	4/31 (12.9%)	0.493	4/31 (12.9%)	0.020 *	4/31 (12.9%)	0.042 *	24/31 (77.4%)	0.159
present	5/25 (20.0%)		10/25 (40.0%)		9/25 (36.0%)		15/25 (60.0%)	
**Lymphovascular invasion**								
absent	1/14 (7.1%)	0.424	1/14 (7.1%)	0.151	0/14 (0%)	0.025 *	1/14 (7.1%)	0.043 *
present	8/42 (19.0%)		13/42 (31.0%)		13/42 (31.0%)		16/42 (38.1%)	
**Distant metastasis**								
absent	9/50 (18.0%)	0.575	13/50 (26.0%)	1.000	12/50 (24.0%)	1.000	36/50 (72.0%)	0.354
present	0/6 (0.0%)		1/6 (16.7%)		1/6 (16.7%)		3/6 (50.0%)	
**Recurrence**								
absent	6/42 (14.3%)	0.676	9/42 (21.4%)	0.304	8/42 (19.0%)	0.274	9/42 (21.4%)	0.019 *
present	3/14 (21.4%)		5/14 (35.7%)		5/14 (35.7%)		8/14 (57.1%)	

^§^ The cut-off values are as follows: miR-429, 0.2-fold; miR-22-3p, 0.5-fold; miR-30e-5p, 0.4-fold; miR-134, 1.5-fold. (*, *p* < 0.05).

**Table 4:** The correlation between HPV16- and HPV16- or 18-infection and miR-22-3p expression.

	**HPV16 Positive**	***p*** **-Value**
miR-22-3p up-regulation ^§^	11/12 (91.7%)	0.021 *
no up-regulation	24/44 (54.5%)	
	**HPV16 or 18 Positive**	***p-*** **Value**
miR-22-3p up-regulation ^§^	12/12 (100.0%)	0.005 *
no up-regulation	25/44 (56.8%)	

^§^ The cut-off value of up-regulation of miR-22-3p is 1.8-fold. (*, *p* < 0.05).

**Table 5:** Multivariate Cox analysis of miR-30e-5p in cervical squamous cell carcinoma.

		Case (n = 56)	Death	Overall Survival (Mon)	Hazard Ratio	*p*-Value
**Age**	<60	49	8	39.2 ± 26.1	**34.14**	0.026 *
	≥60	7	2	35.7 ± 18.0		
**Clinical stage**	I	27	3	46.6 ± 30.7	0.16	0.235
	II/III/IV	29	7	1.6 ± 15.9		
**Nodal metastasis**	absent	31	1	39.7 ± 25.9	0.05	0.219
	present	25	9	37.5 ± 24.7		
**Lymphovascular invasion**	absent	14	0	38.9 ± 27.3	7.11 × 10^4^	0.94
	present	42	10	38.7 ± 24.8		
**Distant metastasis**	absent	50	6	39.8 ± 26.0	**123.76**	0.025 *
	present	6	4	30.4 ± 15.1		
**Recurrence**	absent	42	0	42.7 ± 27.1	1.73 × 10^7^	0.865
	present	14	10	26.8 ± 12.3		
**miR-30e-5p ^§^**	up-regulation	43	6	42.0 ± 26.7	**289.66**	0.040 *
	down-regulation	13	4	28.1 ± 15.7		

^§^ The cut-off value of down-regulation of miR-30e-5p is 0.4-fold (*, *p* < 0.05).

## Data Availability

Not applicable.

## Supplementary Materials

The following supporting information can be downloaded at https://www.mdpi.com/article/10.3390/ijms23105623/s1.
