# Supplementary material for: miR-22-3p and miR-30e-5p Are Associated with Prognosis in Cervical Squamous Cell Carcinoma
Source: Int J Mol Sci. 2022 May 17;23(10):5623. doi: 10.3390/ijms23105623 (PMC9144648; doi:10.3390/ijms23105623)
Supplement: Supplementary file 1 [file ijms-23-05623-s001.zip › ijms-1672829-supplementary.pdf]

Supplementary Files

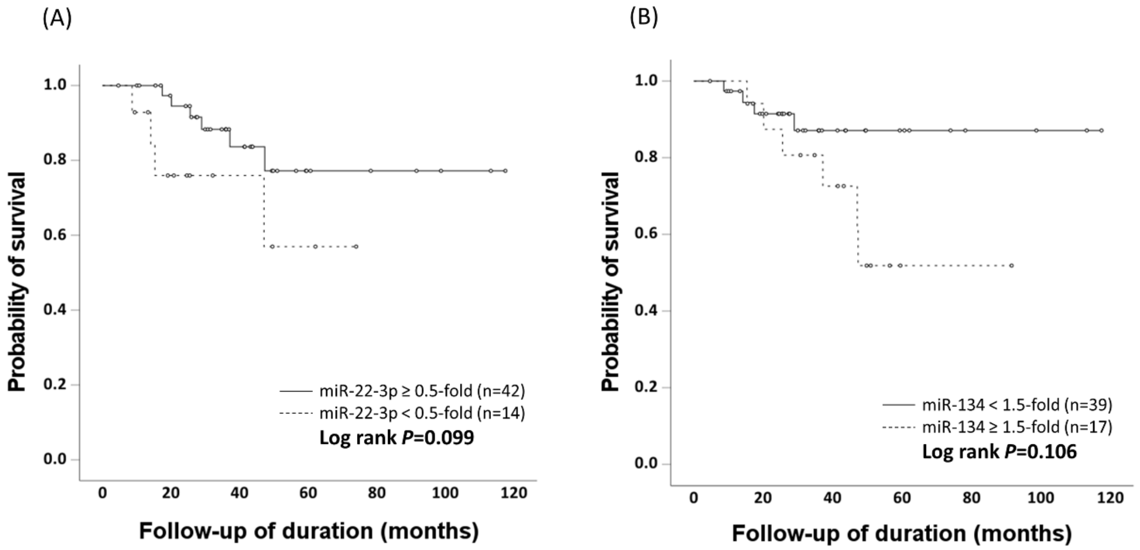

Figure S1. Kaplan-Meier survival curves according to miR-22-3p and miR-134 expression in 56 cases of cervical SqCC. Both miR-22-3p and miR-134 do not show significant difference in OS analysis (downregulation of miR-22-3p, <0.5-fold relative to the normal epithelium (NC) level, log rank=0.099; and up-regulation of miR-134, ≥1.5-fold relative to the NC level, log rank=0.106).

Table S1. The raw Ct values of the four miRNAs; miR-429, miR-22-3p, miR-30e-5p and miR-134 in qRT-PCR.

	miR-429	miR-22-3p	miR-30e-5p	miR-134	RNU48
NC1	35.6194	36.0284	32.9235	38.7864	28.2512
NC2	34.9088	34.5062	31.905	36.5567	28.0654
NC3	35.0792	35.5222	33.0462	40.0402	28.9279
NC4	33.108	32.5424	28.558	35.3352	25.9662
NC5	31.8176	31.9984	28.2134	35.124	25.9693
NC6	31.8434	30.6019	28.0193	33.7342	25.3862
NC7	31.958	30.9524	27.9674	33.6194	25.6319
NC8	31.4093	30.9948	27.6805	33.4887	26.0858
NC9	31.2039	31.3464	27.5375	34.7339	25.9096
NC10	31.6921	32.5901	28.5012	36.2628	26.4863
SC1	35.4753	33.4558	32.0598	37.0972	29.0995
SC2	34.3647	33.7261	30.9392	36.5686	28.2271
SC3	36.5253	32.1762	31.0593	35.4272	29.1032
SC4	35.6502	33.0023	30.8245	36.4153	27.4095
SC5	36.3948	34.4291	32.9173	37.7454	27.9413
SC6	36.7658	35.0731	32.7915	37.1187	28.6151
SC7	34.2492	32.9288	32.1745	36.1594	28.9434
SC8	35.5502	34.6208	32.1178	37.7513	27.7439
SC9	37.3381	35.2128	32.627	39.7753	31.3014
SC10	37.5998	34.3053	31.8896	37.2478	28.758
SC11	37.9254	33.2053	34.675	37.534	33.8586
SC12	35.7365	34.6571	32.0692	36.8814	27.9727
SC13	33.8695	33.7125	31.6509	37.6317	28.5701
SC14	35.5075	33.9484	30.7397	37.6819	27.8992
SC15	36.5944	35.6236	31.4479	37.5159	26.977
SC16	35.7901	33.7757	31.8785	39.4768	28.1803
SC17	34.0229	32.1617	30.4541	36.5288	27.4306
SC18	35.5399	34.1112	32.1383	37.7576	28.6078
SC19	34.8933	33.8795	31.2241	36.7799	27.5466
SC20	36.3161	34.1531	31.8117	36.4544	28.8415
SC21	37.1602	37.0679	32.2451	37.7124	27.2818
SC22	36.6555	35.8797	32.1848	36.4827	29.1135
SC23	33.8795	34.2959	30.6723	36.2581	28.013
SC24	35.3584	35.06	30.8827	38.7138	27.7891
SC25	37.0713	36.776	33.1444	37.046	29.136
SC26	34.057	36.6007	30.4949	38.1788	29.2255
SC27	34.561	35.2636	31.3935	36.8518	29.3621
SC28	37.1145	35.8167	33.0278	38.3018	29.6281
SC29	34.6491	34.649	31.7993	37.1677	28.2865
SC30	34.4681	34.3809	30.6304	35.6724	27.6759
SC31	35.2929	34.9749	31.622	36.2524	28.405
SC32	35.2335	34.3534	30.411	35.4592	27.1257
SC33	34.4037	34.5537	30.7372	35.9118	28.2281
SC34	34.5418	34.1358	31.4159	35.0333	27.9968
SC35	34.9178	32.7735	29.9282	36.9877	28.8369
SC36	34.55	32.7765	31.1598	35.974	27.5771
SC37	34.6352	33.5325	31.5421	36.1326	27.1543

SC38	32.7878	35.004	31.2815	37.5626	28.585
SC39	35.5761	33.9275	31.2467	35.121	27.0385
SC40	35.3531	34.6572	31.9969	36.7403	26.3656
SC41	33.6452	34.4645	31.9726	36.3306	27.4277
SC42	34.2729	33.1531	30.349	34.3513	27.3681
SC43	33.7383	33.4094	28.8688	36.1641	26.4971
SC44	35.5667	35.4266	33.1724	36.5489	27.0157
SC45	36.31	36.7207	30.6364	37.0213	26.9056
SC46	34.5593	35.3229	31.3986	37.0229	26.5615
SC47	34.2796	35.8951	29.9375	36.7316	27.481
SC48	37.2295	35.0969	31.4372	36.0206	27.5863
SC49	32.1307	34.2424	28.1451	37.3768	27.8187
SC50	34.9401	36.471	30.1247	36.1739	26.9582
SC51	34.0884	35.4675	28.8927	35.119	26.2956
SC52	36.2062	35.7569	31.3157	35.4265	28.2071
SC53	32.3572	35.513	28.4821	39.3183	27.9311
SC54	35.4441	34.5361	31.2622	36.4608	27.1503
SC55	36.187	36.5611	32.2848	37.4604	27.757
SC56	36.8786	33.6784	32.9563	37.5315	31.7894
SC57	35.3186	33.5913	31.4287	36.8219	28.1748
SC58	35.3707	33.7652	31.0659	39.0965	28.0657
SC59	39.8837	33.0823	30.5531	42.3547	27.2781
SC60	34.4337	32.1431	29.4809	34.047	26.3728
SC61	34.5531	32.2276	29.7342	37.2904	27.2539
SC62	35.077	32.3587	30.384	36.3579	27.1762
SC63	35.457	32.9293	30.3934	36.3415	27.4601
SC64	36.5454	33.2578	30.7413	37.6306	28.8319
SC65	35.4751	32.0257	30.5624	37.6018	28.1872
	35.02±1.62	34.11±1.47	31.00±1.47	36.79±1.49	27.89±1.33

NC, normal tissue; SC, squamous cell carcinoma; RNU48, control

Table S2. Clinicopathologic characteristics of 56 patients with cervical squamous cell carcinoma, including 29 patients without metastasis and 27 patients with metastasis.

		Patients	Non-metastasis	Metastasis
**Age (yr)**		47.9±11.8	47.8±11.2	48.0±21.6
**HPV**	absent	1	1	0
	low risk	1	1	0
	high risk	54	27	27
**Stage**	I	27	19	8
	II	29	10	15
	III	3	0	3
	IV	1	0	1
**Nodal metastasis**	absent	31	29	2
	present	25	0	25
**Lymphovasuclar invasion**	absent	14	13	1
	present	42	16	26
**Distant metastasis**	absent	50	29	21
	present	6	0	6
**Recurrence**	absent	42	29	13
	present	14	0	14
**Total**		56	29	27
